# Assessment of the direct hospital cost of medical care for patients hospitalized for a stroke in Gabon

**DOI:** 10.11604/pamj.2023.45.95.35702

**Published:** 2023-06-21

**Authors:** Gaëtan Moukoumbi Lipenguet, Edgard Brice Ngoungou, Euloge Ibinga, Prudence Gnamien Amani, Jean Engohang-Ndong, Elsa Ayo Bivigou, Andréa Annick Nsounda, Jérôme Wittwer

**Affiliations:** 1 Bordeaux Health Population, Inserm U1219 University of Bordeaux, 33076 Bordeaux Cedex, France,; 2 Research Unit in Epidemiology of Chronic Diseases and Environmental Health (UREMCSE), University of Health Sciences, Libreville, Gabon,; 3 Department of Epidemiology, Biostatistics and Medical Informatics (DEBIM), University of Health Sciences, Faculty of Medicine, Libreville, Gabon,; 4 Department of Biological Sciences, Kent State University at Tuscarawas, New Philadelphia, United States of America,; 5 Department of Cardiology, *Centre Hospitalier Universitaire de* Libreville, Libreville, Gabon,; 6 Department of Neurology, *Centre Hospitalier Universitaire de* Libreville, Libreville, Gabon

**Keywords:** Stroke, direct cost of health care, Gabon

## Abstract

While the incidence of stroke is increasing in developing countries, resulting in an extremely high economic burden, very few costing studies have been carried out to date. This study aims to measure the direct hospital costs of stroke management in Gabon. The study adopts a retrospective approach, based on a review of patient records in the Neurology and Cardiology Departments of the University Hospital of Libreville (CHUL) between January 2018 and December 2019. It focuses on all patients received for stroke at the CHUL during the study period, regardless of the outcome, analyzing direct hospital costs. Three hundred and thirteen (313) patients were admitted during the period in question, 72.52% in neurology and 27.48% in cardiology. The average age was 58.44 (±13.73 years). Fifty-six percent (56.23%) had health coverage. Ischemic stroke was more common than hemorrhagic stroke, at 79.55% and 20.45%, respectively. The average expenditure per patient was estimated at 619,633 CFA francs (€944.62). From the point of view of social security coverage, the average out-of-pocket expense per patient was 147,140 CFA francs (€224.31), for a reimbursement of 422,883 CFA francs (€644.68). The average direct cost of stroke is very high for both patients and administrations. This argues for the implementation of prevention programs for the disease. The results of this study may be useful for work on the efficiency of such programs.

## Introduction

Stroke, commonly referred to as “cerebrovascular accident”, is defined by the World Health Organization (WHO) as a neurological deficit of “rapid” onset, lasting more than 24 hours, associated with focal or global cerebral dysfunction which can be fatal, and whose apparent cause is vascular [1]. It results from the interruption of blood flow to the brain, usually when a blood vessel bursts or is blocked by a clot, stopping the supply of oxygen and nutrients and causing damage to brain tissue. The condition occurs in both developed and developing countries. While the prevalence of the disease is unknown in many developing countries, the number of cases is high. According to WHO data in 2010, 68.6% of new stroke cases, 70.9% of stroke deaths, and 77.7% of years of life lost due to residual disability or stroke-related death occurred in both low- and middle-income countries [2]. Despite the advances made in recent years in both diagnostic and therapeutic techniques, we have witnessed an increase in incidence of the disease [3,4]. Modern management techniques currently being developed appear to be inaccessible in low-income countries. Thrombolysis or endovascular thrombectomy, recommended in the emergency management of stroke, are not readily accessible in most African countries. The burden of this pathology is felt not only in health terms but also economically. In terms of health, management of the disease requires human, material, and financial resources to improve the patient´s vital and functional prognosis. Numerous studies conducted across the globe show that the pathology is very costly, presenting a heavy burden not only for health systems and the economy, but also for the patient [5-7]. The cost of stroke management varies greatly from country to country. In France, depending on the source and nature, the cost of incident cases over one year varies between 5,142,384 CFA francs (€7,839) for a mild ischemic stroke and 27,182,672 CFA francs (€41,437) for a severe hemorrhagic stroke [6]. In China, the average cost of hospitalization for an ischemic stroke in 2010 was 10,689 Yuan (€1,368.2 or 897,500 CFA francs) and for a hemorrhagic stroke it was 13,089 Yuan (€1,675.4 or 1,099,016 CFA francs) [8]. In Africa, the average cost of a stroke varies from one country to another, and the calculation methods also vary between studies. In Senegal, Touré K *et al*. estimated a hospital cost of 78,426 CFA francs (€120) per patient [5]. Adoukounou *et al*. study in Benin estimated the average cost of stroke management at 316,810 CFA francs (€483) [7]. In Cameroon, Mapoure *et al*. estimated the average cost for an ischemic stroke at 666,741 CFA francs, i.e., €1,016.4, and a hemorrhagic stroke at 771,199 CFA francs, i.e., €1,175.6 [9]. While overviews of stroke management costs exist for some countries, other countries have no such studies. To our knowledge, no research to date has evaluated the cost of managing this condition in Gabon, hampering cost-effectiveness studies on stroke prevention programs. The present study therefore, evaluates the direct hospital costs of stroke management in the neurology and cardiology departments at the University Hospital of Libreville from the point of view of all funders (patients, health insurance, State). We performed a systematic and exhaustive evaluation of the costs based on all the patients' files over a two-year period.

## Methods

**Study setting:** the present study was conducted in the cardiology and neurology departments of the University Hospital of Libreville (CHUL) in Gabon. The CHUL is one of the three university hospitals in the country and is the only one to have a neurology and a cardiology service. One of the other two university hospitals specializes in trauma care, while the third specializes in maternity care and childcare. Other types of patients are treated at the University Hospital Center, which covers a little over 60% of public-sector hospital interventions. As far as treatment of cardiovascular and neurovascular diseases is concerned, the CHUL is the reference structure in Gabon. It has a surface area of 22,000 m^2^, with a capacity of 696 beds, and it serves a population of approximately 937,105 inhabitants. The CHUL has nine hospitalization units, a pharmacy, a laboratory, a Magnetic Resonance Imaging (MRI) unit, eight operating theaters including one specialized in cardiology, an intensive care unit, and a neuropsychology unit. The choice of these services and this center for the present study was motivated by its resources listed above.

**Type of study:** our paper is based on a retrospective study conducted at the University Hospital of Libreville in Gabon over a 24-month period from January 2018 to December 2019. Data were collected from the paper-based patient records of 313 patients admitted to one of the CHUL´s neurology or cardiology departments for stroke, without specifics. The characteristics of the study population is presented in the results section of the study.

**Data collection:** data collection was carried out retrospectively by analyzing the patient files archived in the two departments mentioned above, on the one hand, and the pharmacies on the other hand, with further information derived from reference frameworks for the price of medical acts and medications. A collection sheet was designed to this end. Complete records (approximately 92% of the records) were included in the study. As indicated above, three sources of data were used to gather the study data.

**Data from patient records in paper format:** the patient records in paper format served as data collection media for sociodemographic characteristics (age, sex, socio-professional status, insurance status, etc.), the patients´ clinical status (type of stroke, severity of the disease, etc.), prescriptions for the acts administered (radiology examination, laboratory tests, and medication), and the length of hospitalization for each patient.

**Health coverage pricing data:** the medical acts were priced on the basis of healthcare structure price references produced by the social security in collaboration with the Gabonese Ministry of Health in 2013 [10] and revised in 2018 [11]. The procedures covered in the study were consultation, imaging and laboratory examinations, and hospital stays.

**Drug and medical device price data:** the cost of pharmaceutical products (drugs and medical devices) was calculated on the basis of current market prices, whether or not reimbursed by social security, as collected from pharmacies. For items reimbursed by social security, a rate covered by the latter was applied to the market price for the years included in the study in order to determine the out-of-pocket expenses for patients and health insurance. The main social security or health insurance system is called the National Health Insurance and Social Guarantee Fund (CNAMGS). It covers almost the whole Gabonese population. It is a compulsory insurance scheme that is applicable on Gabonese territory. It reimburses patients' health care at a conventional rate ranging from 50% to 80%, depending on the procedure performed on the patient. Patients with long-term illnesses (ALD) are reimbursed at a rate of 95%. The remaining cost for such patients is thus 5% of the overall cost of their treatment.

**Expenditure per patient:** expenditure per patient encompasses the aggregation of the cost of hospital days multiplied by the length of stay, the cost of laboratory tests, the cost of drugs, the cost of medical equipment, the cost of consultation on admission, and the cost of examinations in specialized services. The hospitalization fee set by the establishment includes the cost of maintaining the premises, utility bills, and hotel accommodation.

**Determining the cost of salaries for professionals:** the labor costs of medical personnel (doctors´ and nurses´ salaries) were estimated on the basis of the salaries set by the Gabonese government. A global amount for salaries over two years was obtained by considering the salary per category of personnel multiplied by the number of caregivers in the two services, for a total of 196,800,000 CFA francs (€300,000). Stroke management represents 9.62% of the neurology and cardiology departments´ activities at the CHUL. To estimate the cost of stroke care, the percentage of stroke activity was applied to the overall wage bill of health professionals in the two departments for the two years investigated. Thus, the amount paid to professionals for stroke management was 17,712,000 CFA francs (€27,000). For cost per patient, the estimated amount for stroke management was based on all of the patients hospitalized for stroke in both departments during the study period (313 patients). We hypothesized a homogeneous patient management time regardless of the type of stroke, i.e., 56,587 francs (€82.3) per patient. We did not have the means to be more precise regarding cost per patient as a function of the time spent in care. The cost variables are expressed in CFA francs in 2019. The conversion rate into euros is €1 for 655.975 CFA francs.

**Economic approach:** we adopted a bottom-up approach to data collection. This involves an individual approach to each patient´s expenses via a questionnaire in which all the expenses are systematically recorded. We limited this to an evaluation of the direct hospital cost, in other words, the sum of costs by category (consultations, transport, treatment, paraclinical examinations, rehabilitation, hospitalization costs, other costs during hospitalization). The calculation principle involved combining all the costs in order to determine the average expenditure per patient.

**Data management and analysis:** the data collected and collated in the database were analyzed using R software, version 3.6.1. Quantitative variables were expressed as means with their standard deviations, and qualitative data as percentages. Means and standard deviations were calculated for all the cost variables. To search for a possible link between the dependent variable and the independent variables, a univariate analysis was performed with a significance level of 5%. For this analysis, the cost variables were classified as appropriate, and the average cost was compared between the categories of independent variables. Sensitivity analysis was performed using a bootstrap-type resampling method of 1,000 draws.

**Ethics approval:** the study performed here was primarily observational, retrospective, and based exclusively on medical records. It was non-invasive and carried no physical, mental, or emotional risk to the patient. It did not involve human or animal experimentation. Our study was therefore exempt from ethics approval procedure currently applicable in Gabon. Nevertheless, in compliance with Gabon research protocols, two research authorizations were obtained for the collection of information on medical records. One was issued by the General Secretary of the Ministry of Health (N° 002395/MS/SG/PSNIS) and the second was issued by the Chief Executive Officer of the CHUL (N° 0078/MS/CHUL/DG/DGA).

## Results

The study provides an exhaustive two-year (2018 and 2019) description of the hospital costs of patients admitted to the CHUL for both hemorrhagic stroke and ischemic stroke.

**Patient demographics:** three hundred and thirteen (313) patients were registered, 227 (72.5%) in neurology and 86 (27.48%) in cardiology. The average age of the patients was 58.4 years (±13.7 years). More specifically, the average age for men was 57.8 years (±12.8 years) and for women it was 59.2 years (±14.8 years). The male sex was predominant, with 173 men (55.3%) against 140 women (44.7%), i.e., a sex ratio of 1.2 in favor of men. Fifty-six percent (56.2%) of patients had health insurance, compared with 43 percent (43.7%) who did not. Ischemic stroke was more common than hemorrhagic stroke, at 79.5% and 20.4%, respectively. The mean length of hospitalization for our study period was 10.52 days (±8.2 days). Table 1 presents the characteristics of our study population.

**Table 1:** sociodemographic characteristics of patients

Variable	Patient set N= 313	Insured patient N=176 (56.2%)	Uninsured patient N= 137 (43.8%)
Age, mean (SD)	58.4 (13.7)	58.8 (14.8)	57.9 (12.3)
**Gender, N (%)**			
Male	173 (55.3)	83 (47.2)	90 (65.7)
Feminine	140 (44.7)	93 (52.8)	47 (34.3)
**Medical history, N (%)**			
HTA	244 (77.9)	141 (80.1)	103 (75.2)
Diabetes	262 (83.7)	145 (82.4)	117 (85.4)
Hypercholesterolemia	301 (96.7)	170 (96.6)	131 (95.6)
Alcohol	163 (52.1)	94 (53.4)	69 (50.4)
**Service, N (%)**			
Neurology	227 (72.5)	120 (68.2)	107 (78.1)
Cardiology	86 (27.5)	56 (31.8)	30 (21.9)
**Type of stroke, N (%)**			
Ischemic	249 (79.5)	140 (79.5)	109 (79.6)
Hemorrhagic	64 (20.5)	36 (20.5)	28 (20.4)
**Become, N (%)**			
Home exit	265 (84.7)	153 (86.9)	112 (81.8)
Transferred	22 (7.0)	11 (6.3)	11 (8.0)
Deceased	23 (7.4)	12 (6.8)	11 (8.0)
Escaped	3 (0.9)	0 (0)	3 (2.2)
**Marital status, N (%)**			
Single	97 (31.0)	56 (31.8)	41 (29.9)
Paxed	40 (12.8)	24 (13.6)	16 (11.7)
Married	128 (40.9)	67 (38.1)	61 (44.5)
Divorced	3 (1.0)	2 (1.1)	1 (0.7)
Widower (d)	45 (14.4)	27 (15.3)	18 (13.1)
**Status, N (%)**			
NP	92 (29.4)	58 (33.0)	34 (24.8)
Student / Student	1 (0.3)	1 (0.6)	-
Unemployed	19 (6.1)	14 (8.0)	5 (3.6)
Medium frame	48 (15.3)	31 (17.6)	17 (12.6)
Senior	15 (4.8)	10 (5.7)	5 (3.6)
Worker	50 (16.0)	12 (6.8)	38 (27.7)
Official	9 (2.9)	6 (3.4)	3 (2.2)
Trader	6 (1.9)	2 (1.1)	4 (2.9)
Retirement	73 (23.3)	42 (23.9)	31 (22.6)
Average length of stay (SD)	10.5 (8.2)	10.7 (7.9)	10.2 (8.6)

Sociodemographic characteristics of our study population received at CHUL between 2018 and 2019

**Cost of ischemic stroke management:** the average length of stays for ischemic stroke management was 10.4 days (±8.7 days). Sixty-eight percent (68%) of ischemic stroke patients were hospitalized in the neurology department. On average, patients spent 557,491 CFA francs (€849.8) for an ischemic stroke. Uninsured patients had an average expenditure of 527,638 CFA francs (€804.3). Those covered by social security spent an average of 141,434 CFA francs (€215.6), and the remaining cost to the health insurance company was 420,140 CFA francs (€640.4). Total expenditure for ischemic stroke was 153,877,723 CFA francs (€234,569.7), as presented in Table 2.

**Table 2:** sociodemographic and clinical characteristics correlated with the cost of stroke management

Characteristics	Uninsured patients		Insured patients			
	Mean (SD)	p-value	Insurance share		Remainder to pay	
			Mean(SD)	p-value	Mean(SD)	p-value
**Age**						
<=45	556,092 (224,329)	0.6	438,335 (223,090)	0.7	153,520 (89,158)	0.7
(45 – 55)	512,756 (155,263)		402,841 (136,994)		138,323 (61,373)	
(55 – 65)	563,872 (316,390)		400,434 (126,685)		146,782(82,143)	
>65	500,015 (210,808)		398,204 (179,685)		136,989 (70,056)	
**Sex**						
Feminine	510,048 (199,254)	0.4	141,348 (72,062)	0.7	141,348 (72,062)	0.7
Male	543,323 (254,429)		145,359 (80,119)		145,359 (80,119)	
**Type of stroke**						
Hemorrhagic	548,528 (22,8977)	0.6	419,886 (152,979)	0.6	150,260 (80,169)	0.5
Ischemic	527,638 (239,530)		404,042 (170,734)		141,434 (74,797)	
**Income-generating activity**						
Without activity	517,769 (224,883)	0.4	400,290 (174,701)	0.4	140,274 (77,020)	0.4
In activity	545,440 (248,410)		420,140 (152,223)		148,692 (73,736)	
**Co-morbidities**						
No co-morbidities	519,858 (251,290)	0.8	401,924 (144,163)		151,247 (77,221)	0.7
Comorbidity	520,212 (184,633)		395,944 (159,306)	0.6	137,515 (72,823)	
Two or more	539,568 (259,389)		416,157 (176,600)		145,896 (78,069)	

Presentation of average stroke management costs correlated with sociodemographic characteristics; costs expressed in CFA Francs (1euro = 655.975 CFA Francs)

**Cost of hemorrhagic stroke management:** the mean length of stays for hemorrhagic stroke was 10.7 days (±5.5 days). Eighty-nine percent (89%) of hemorrhagic stroke patients were hospitalized in the neurology service. Patients not affiliated to the social security system spent an average of 548,528 CFA francs (€836.2) per person for their care. The remaining cost per insured patient was 150,260 CFA francs (€229.0) and health insurance reimbursement to the care facilities amounted to 419,886 CFA francs (€640.1). Consequently, the total cost of care for patients hospitalized for hemorrhagic stroke was 40,067,522 CFA francs (€61,078.5), as presented in Table 2.

**Figure 1:**
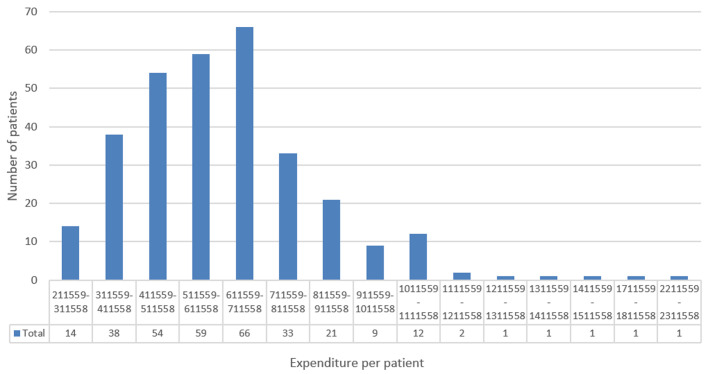
distribution of expenditure per patient; amplitude 100,000

**Cost of overall stroke management:** in general, the average length of stays in hospital was 10.5 days (± 8.2 days) for a stroke episode. The average cost of stroke management during our study period is estimated at 619,633 CFA francs (€944.6). Uninsured patients spent an average of 545,163 CFA francs (€831.0). For insured patients, the expense was 147,140 CFA francs (€224.3) per patient on average. The amount reimbursed to the facility by health insurance is estimated at 422,883 CFA francs (€644.64) per patient. More than half the patients had spent between 411,559 CFA francs and 711,559 CFA francs (Figure 1). The highest cost item in patient care is that of hospitalization, followed by imaging examinations, representing 37.6% and 37.1% of total expenditure, respectively (Table 3). In terms of prescriptions, computed tomography (CT) represents 41.06% of imaging examination-related expenditure, followed by cardiac ultrasounds at 20.45%. In laboratory tests, blood ionograms represent 20.38% of expenditure, followed by HIV serology which represents 20.02% of expenditure related to laboratory tests (Table 4). A multivariable linear regression was used to verify the relationship between the cost of stroke management and the different explanatory variables (sociodemographic factors, medical history, variations in length of hospitalization, and type of stroke). No variable explained the cost variation in the management of stroke patients in the neurology and cardiology departments at the CHUL. A bootstrap-type resampling method of 1,000 draws was carried out in order to represent the expenditure per patient on an abstract sample. The results of the simulation show expenditure per patient at 619,617 FCFA (€944.53) and a 95% CI of [597080 - 644714].

**Table 3:** average cost of care by item of expenditure, per insured and uninsured patient

Nature of care	Global (% of expenditure)	Uninsured patients mean (N=137)	Insured patients			All patients	
			Insured patients	Remainder to pay (N=176)	Insurance share (N=176)	Mean (N=313)	Extreme
Consultation	3,130,000 (1.6)	10,000	10,000	2,000	8,000	10,000	[10,000 – 10,000]
Hospitalization	65,880,000 (34.2)	204,963	214,772	42,954	171,818	210,479	[20,000 – 1,900,000]
Laboratory tests	13,862,000 (7.2)	45,583	43,278	8,911	34,367	44,287	[0 – 685,000]
Imaging exams	64,844,000 (33.6)	195,729	216,073	63,157	152,915	207,169	[20,000 – 709,000]
Physiotherapy	17,280,000 (9.0)	58,467	52,670	16,971	35,698	55,207	[0 – 90,000]
Medications	10,015,562 (5.2)	30,418	33,228	13,144	20,283	31,998	[0 – 146,540]
Salary	17,712,000 (9.2)	-	-	-	-	56 587	[300,000- 2,500,000]

Average expenditure, taking into account the positions in the chain of care of the patient; costs expressed in CFA Francs (1 euro = 655.975 CFA Francs)

**Table 4:** unit cost and average cost by type of exam

Exam type	Number of acts (%)	Unit cost	Patient set	Insured patients	Uninsured patients
**Imaging exam (% of imaging exam expense)**					
MRI (7.77%)	19 (6.07)	260,000	16,102	16,250	15,912
CT (41.06%)	256 (81.79)	104,000	85,060	86,272	83,503
ECG (7.22%)	234 (61.34)	20,000	14,952	15,681	14,014
Cardiac ultrasound (20.45%)	204 (65.18)	20,000	42,364	43,210	41,277
Ultrasound vessels neck (14.68%)	136 (43.45)	70,000	29,635	31,022	29,635
Angio-MRI CT Angiography (8.82%)					
**Laboratory tests (% of laboratory test expense)**	22 (7.03)	260,000	18,274	23,636	11,386
Blood sugar (5.30%)	245 (78.27)	3,000	2,348	2,301	2,408
Blood formula count (13.96%)	258 (82.43)	7,500	6,182	5,923	6,514
LDL cholesterol (4.48%)	207 (66.13)	3,000	1,984	2,028	1,927
HDL cholesterol (4.39%)	203 (66.13)	3,000	1,945	1,926	1,970
Triglyceride (3.94%)	182 (58.15)	3,000	1,744	1,653	1,861
Ionogram (20.38%)	226 (72.2)	12,500	9,025	8,735	9,397
Urea (5.76%)	274 (87.54)	3,500	2,549	2,068	3,167
Creatinine (7.07%)	280 (89.46)	3,500	3,130	3,062	3,218
TP (4.57%)	181 (57.83)	3,500	2,023	2,147	1,864
Tck (3.59%)	171 (54.63)	3,500	1,587	1,670	1,481
HIV serologies (20.02%)	185 (59.11)	15,000	8,865	8,778	8,978
TPHA VDRL serologies (6.55%)	192 (61.34)	7,500	2,899	2,982	2,791

Amount applied per type of examination, laboratory, and imaging; examinations on the table are those prescribed to patients during their hospital stay; costs expressed in CFA Francs (1euro = 655.975 CFA Francs)

## Discussion

The present study allowed us to evaluate the direct cost of stroke management in the neurology and cardiology departments of the University Hospital of Libreville in Gabon. This evaluation is necessary since there is a lack of knowledge regarding the costs related to management of strokes whose prevalence is increasing. It was carried out from the perspective of all the funders (patients, health insurance, and the State). The costs evaluated in the study included all the patient´s medical care, his or her hospital stay, and remuneration of the health care personnel assigned to the services selected during the study period. Indirect costs such as patient transport and loss of income for both the patient and the accompanying person were not included. Three hundred and thirteen patients were included in the study, whose average age was 58.4 years (±13.7 years). The mean age of the study population is similar to that in the study by Adoukounou in Benin [7], but is lower than that found in a study conducted in Cameroon (60 ± 12.5 years) [9]. The predominant gender was male, with 55% of men. This male preponderance has been found in most African studies [7,9], except that of Ogun SA *et al*. [12], where women made up most of the study population (54.9%). Ischemic stroke was predominant (79.5%) in our study compared to hemorrhagic stroke. This result is similar to most of the literature on the costs of stroke in Africa [5,13-15], except for the study by Ogun *et al*. [12] in southern Nigeria, which found a predominance of hemorrhagic stroke (51%). This may be explained by the subject inclusion method used since the patients included in the study did not have an imaging examination such as a CT, but were classified in one or other of the types of stroke according to their clinical signs. Regarding clinical management, there was little difference in the mean length of hospital stays according to the stroke type. For ischemic stroke, it was 10.4 days (±8.7 days), while (±5.5 days) for hemorrhagic stroke (p-value =0.7) it was 10.7 days. The direct cost of ischemic stroke management was 619,633 CFA francs (€944.6) per patient on average, while that of hemorrhagic stroke was 626 055 CFA francs (€954.3). With no distinction made between the type of stroke, cost per patient was 617,982 CFA francs (€942.0). The results of this study are comparable to those of studies performed in some African countries, with the exception of the study by Touré *et al*. in Senegal [5] which presents a relatively low average cost compared to the other studies, estimating the average cost of stroke management at 78,426 F CFA (€119.5). This low cost could be explained by the fact that cerebral CT scans were not systematically performed to diagnose the ischemic or hemorrhagic nature of the stroke. Only 60% of patients had radiology examinations since they were considered too expensive. Two studies have been conducted on the cost of stroke management in Togo, with both presenting practically the same costs [13,16]. In 2010, Guinhouya KM *et al*. estimated the cost of stroke management at Sylvanus Olympio University Hospital in Lomé at an average of 445,512 CFA francs € (679.1) per patient [13], while in 2016, Balaka A *et al*. calculated the average cost at 312,245 CFA francs (€475.9) per patient for an average stay of 18.6 days [16]. In a study of 78 patients in Benin, Adoukonou *et al*. estimated the average cost per patient at 316,810 CFA francs (€482.94) for a stroke episode [7]. The highest average cost was found in the study by Njankouo *et al*. in Cameroon, where the typical patient spent 802,355 CFA francs (€1223.1) [9]. This difference could be explained by the fact that the study not only presents direct and indirect costs, but also includes management of patients outside the health care facility as well as the cost of stroke management by traditional physicians.

It should be noted, however, that stroke management in traditional medicine has often not been evaluated in several African countries, although patients sometimes use this medicine before going to the hospital. In their study in Cameroon, Njankouo *et al*. aimed not only to present the high cost of stroke management, but also made a plea to the country's authorities to set up a health insurance scheme to help patients in their care [9]. From the point of view of health coverage in our study, the average reimbursement to health care facilities was 422 883 CFA francs (€644.64) per patient. The remaining cost for the insured patient was 147,140 CFA francs (224.3 €), which represents one-third of the average cost for patients not covered by social security. Although this coverage remains high overall, it is less burdensome for an insured patient. This result cannot be compared with the findings of the research mentioned above as the countries investigated did not have health insurance at the time the studies were conducted. Comparison of the costs of care in different studies is difficult given the disparities linked to the realities of each individual country. Some countries, especially those in sub-Saharan Africa, face multiple challenges with their health systems, their technical facilities, and sometimes the absence of specialists, hampering management of the disease. Stroke management is extremely expensive, both for patients who must sometimes bear the cost alone, and for national health care systems. Studies on the direct costs of stroke patients provide valuable information on the high price tag of strokes in both developing and developed countries. In some countries, although there is a health coverage system that can reduce patients´ out-of-pocket expenses, the cost of a stroke remains high. Gabon has a health coverage system, but the cost of treating a stroke is nonetheless more than the minimum wage received by the lowest paid workers, i.e., 150,000 CFA francs (€228.6). Patients who are not affiliated to the social security system spend an average of 545,163 CFA francs (€831), a cost 3.6 times higher than the minimum wage in Gabon. In Togo, the cost of stroke care is 19 times higher than the minimum monthly salary of civil servants, i.e., 35,000 CFA francs (€53.3) [16]. The guaranteed interprofessional minimum wage (SMIG) in Cameroon is 28,216 CFA francs (€43) as of 2018. The average cost per patient in Cameroon is 802,355 CFA francs (€1223.1) which is 28.4 times higher than the SMIG [9]. In comparison with the different minimum wages in African countries, stroke management is ostensibly very expensive on the African continent.

**Limitations:** the study has several limitations. The costs discussed here do not take into account direct costs related to patient transport, or indirect costs related to the loss of income of caregivers during the period of hospitalization. They also do not include the costs of post-hospitalization care for patients.

## Conclusion

Studies on the direct or indirect costs of stroke management in developing countries are rare. In the case of Gabon, this study is one of the first to evaluate the direct hospital costs of patients hospitalized for stroke. It provides an estimate of the economic burden of this pathology in terms of the hospital cost of stroke management. Although it has limitations since it focuses on direct costs, the study nonetheless shows that the cost of stroke management in Gabon is very high for patients, regardless of their coverage status, and for the State in terms of the reimbursement of procedures and the remuneration of health professionals. Hospitalization and imaging examinations appear to be the biggest expenditure items. A major step forward would be to reduce the cost of imaging examinations (MRI and CT) borne by the patient, currently around 50%. The results of this study suggest that government involvement to alleviate patients´ expenses would be a welcome development as treatment remains very costly in countries where populations live below the poverty line. The implementation of prevention programs to fight against the pathology would also be welcome.

## References

[1] World Health Organization (2000). World health report. Geneva.

[2] World Health Organization (2010). Organisation Mondiale de la Santé. Rapport d´activité.

[3] Connor M D, Walker R, Modi G, Warlow CP (2007). Burden of stroke in black population in sub-Saharan Africa. Lancet Neurol.

[4] Sagui E (2007). Les accidents vasculaires cérébraux en Afrique subsaharienne. Med Trop (Mars).

[5] Touré K, Ndiaye NM, Sène Diouf F, Ndiaye M, Diallo AM, Ndao AK (2005). Evaluation du coût de prise en charge des accidents vasculaires cérébraux à Dakar, Sénégal. Med Trop (Mars).

[6] De Pouvourville G (2016). Coût de la prise en charge des accidents vasculaires cérébraux en France. Arch Cardiovasc Dis.

[7] Adoukonou T, Kouna-Ndouongo P, Codjia JM, Covi R, Tognon-Tchegnonsi F, Preux PM (2013). Cout direct hospitalier des accidents vasculaires cérébraux à Parakou au nord du Benin. Pan Afr Med J.

[8] Wei JW, Heeley EL, Jan S, Huang Y, Huang Q, Wang JG (2010). Variations and determinants of hospital costs for acute stroke in China. PLoS One.

[9] Njankouo YM, Tegue CK, Kouna PE, Namme HL, Sone AM, Kongnyu AN (2014). Coût des Accidents Vasculaires Cérébraux à l´Hôpital Général De Douala. Health Sci. Dis.

[10] Caisse Nationale d´Assurance Maladie et de Garantie Sociale (2015). Liste des médicaments remboursables par CNAMGS: Edition 2015.

[11] Caisse Nationale d´Assurance Maladie et de Garantie Sociale (2018). Liste des médicaments remboursables par CNAMG: Edition 2018.

[12] Ogun SA, Ojini FI, Ogungbo B, Kolapo KO, Danesi MA (2005). Stroke in South West Nigeria: a 10-year review. Stroke.

[13] Guinhouya KM, Tall A, Kombate D, Kumako V, Apetse K, Belo M (2010). Cost of stroke in Lomé. Sante.

[14] Sène Diouf F, Basse AM, Ndao AK, Ndiaye M, Touré K, Thiam A (2006). Pronostic fonctionnel des accidents vasculaires cérébraux en Pays en voie de développement : Sénégal. Ann Readapt Med Phys.

[15] Sagui E, M'Baye PS, Dubecq C, Ba Fall K, Niang A, Gning S (2005). Ischemic and hemorrhagic strokes in Dakar, Senegal: a hospital-based study. Stroke.

[16] Balaka A, Tchamdja T, Djagadou KA, Assane H, Némi KD, Djibril MA Medical direct cost of hospital admission for cerebrovascular accident on medical recovery at the Sylvanus Olympio teaching hospital of Lomé. Open J Intern Med.

